# Supplementary material for: The role of hypothyroidism in cirrhosis pathogenesis: A retrospective cohort study and multi-omics integration analysis
Source: PLoS Genet. 2025 Nov 7;21(11):e1011947. doi: 10.1371/journal.pgen.1011947 (PMC12611128; doi:10.1371/journal.pgen.1011947)
Supplement: S1 Checklist — (DOCX) [file pgen.1011947.s016.docx]

**STROBE-MR checklist of recommended items to address in reports of Mendelian randomization studies**^1^ ^2^

| **Item No.** | **Section** | **Checklist item** | **Page No.** | **Relevant text from manuscript** |
| --- | --- | --- | --- | --- |
| 1 | **TITLE and ABSTRACT** | Indicate Mendelian randomization (MR) as the study’s design in the title and/or the abstract if that is a main purpose of the study | 2 | “To assess genetic correlation, we applied linkage disequilibrium score regression, followed by bidirectional Mendelian randomization to explore potential causal relationships. ”  “Finally, we performed molecular docking and phenome-wide Mendelian randomization to identify potential therapeutic compounds targeting the prioritized genes.” Described in Abstract. |
|  | **INTRODUCTION** |  |  |  |
| 2 | **Background** | Explain the scientific background and rationale for the reported study. What is the exposure? Is a potential causal relationship between exposure and outcome plausible? Justify why MR is a helpful method to address the study question | 4-6 | **Scientific background and rationale:** “Liver cirrhosis, the 11th leading global cause of mortality, represents the terminal stage of chronic liver injury marked by hepatic encephalopathy and portal hypertension. While viral hepatitis, alcohol-related liver disease, and non-alcoholic fatty liver disease are primary etiologies, emerging evidence implicates hypothyroidism as a key modifier of cirrhosis progression. Hypothyroidism, affecting 1-7% of adults depending on age and iodine status, is predominantly caused by iodine deficiency and autoimmune disorders. Critically, hypothyroidism exacerbates hepatic fibrosis and metabolic dysfunction, establishing a bidirectional thyroid-hepatic axis: thyroid hormones regulate hepatic lipid metabolism by promoting mitochondrial β-oxidation and cholesterol efflux, while impaired hepatic function reduces peripheral thyroxine (T4) to triiodothyronine (T3) conversion, creating a self-reinforcing pathogenic cycle. This pathological synergy is evidenced by the correlation between low serum T3 levels and cirrhosis severity, as well as preclinical models demonstrating hypothyroidism-induced hepatic steatosis and fibrosis via disrupted autophagy and lipid turnover. These findings suggested that hypothyroidism may increase the risk of cirrhosis through multiple mechanisms.”  **What is the exposure?**  When exploring the causal relationship between hypothyroidism and liver cirrhosis, "exposure" refers to hypothyroidism; while when exploring the potential relationship with 675 different phenotypes from various systems throughout the body, "exposure" refers to these 675 distinct phenotypes.  **Is a potential causal relationship between exposure and outcome plausible?**  Yes, the reasons were described in the first paragraph of the introduction.  **Justify why MR is a helpful method to address the study question**  “Large-scale clinical cohorts provide direct evidence for associations between hypothyroidism and cirrhosis, yet such observational studies cannot elucidate the biological mechanisms driving this association. Therefore, to reveal their deeper and more complex interaction mechanisms, integrating multidimensional functional evidence is essential. Fortunately, the emergence of a multi-omics framework provides the robust tools to dissect these complex interactions. For instance, genome-wide association studies (GWAS) enable the identification of genetic variants, while leveraging their random allocation effectively mitigates confounding in causal inference” |
| 3 | **Objectives** | State specific objectives clearly, including pre-specified causal hypotheses (if any). State that MR is a method that, under specific assumptions, intends to estimate causal effects | 6,37 | **State specific objectives clearly:**  “Therefore, this study aimed to systematically evaluate the causal relationship between hypothyroidism and cirrhosis, identify key genes and pathways mediating this association, elucidate the underlying cell-type-specific molecular mechanisms, and ultimately, to screen for and validate potential therapeutic targets.”  **State that MR is a method that, under specific assumptions, intends to estimate causal effects:**  “The MR analysis relied on three core assumptions: the relevance assumption requiring a strong association between instrumental variable (IV) and exposure; the independence assumption ensuring IVs are unaffected by confounders; and the exclusion restriction assumption stipulating that IVs influence the outcome solely through the exposure.” |
|  | **METHODS** |  |  |  |
| 4 | **Study design and data sources** | Present key elements of the study design early in the article. Consider including a table listing sources of data for all phases of the study. For each data source contributing to the analysis, describe the following: | 33-34 | “GWAS summary statistics for hypothyroidism and cirrhosis were obtained from four independent cohorts to support a two-stage discovery and validation design. For the discovery phase, this study utilized data from the UK Biobank for hypothyroidism, and a meta-analysis by Ghouse et al. for cirrhosis. For subsequent replication, independent GWAS data for both traits were sourced from the FinnGen consortium ***(S1 Table)***.” |
|  | a) | Setting: Describe the study design and the underlying population, if possible. Describe the setting, locations, and relevant dates, including periods of recruitment, exposure, follow-up, and data collection, when available. | 33-34 | The samples were sourced from UK Biobank, and the meta-analysis was conducted by Ghouse et al. And the FinnGen consortium. The study design is presented in **Supplementary Figure**. |
|  | b) | Participants: Give the eligibility criteria, and the sources and methods of selection of participants. Report the sample size, and whether any power or sample size calculations were carried out prior to the main analysis | 38 | The data of the research subjects has been described **“GWAS resource” section** and ***S1 table***. We calculated the F statistic to determine the statistical power of the SNPs:“Single Nucleotide Polymorphisms (SNPs) with F-statistics < 10, calculated as *F* = $\frac{R^{2}\times(N-1-k)}{(1-R^{2})\times k}$ where 𝑅^2^ is the variance explained by IVs, *N* is the sample size, and *k* is the number of IVs, were excluded to minimize weak instrument bias” |
|  | c) | Describe measurement, quality control and selection of genetic variants | 38 | “Genetic instruments for each direction were selected based on genome-wide significance (*P* < 5 × 10⁻⁸) and refined using LD clumping thresholds (*R*² = 0.001, clumping distance = 10,000 kb) to ensure independence. Single Nucleotide Polymorphisms (SNPs) with F-statistics < 10, calculated as *F* = $\frac{R^{2}\times(N-1-k)}{(1-R^{2})\times k}$ where 𝑅^2^ is the variance explained by IVs, *N* is the sample size, and *k* is the number of IVs, were excluded to minimize weak instrument bias. To further ensure the correct causal direction, we applied Steiger filtering, removing any IVs that explained more variance in the outcome than in the exposure (69). To rigorously uphold the core assumptions of MR, we coned a comprehensive screening for potential horizontal pleiotropy. First, we directly screened all IVs against the outcome and removed any variants associated with it that surpassed a significance threshold of *P* < 1 × 10⁻⁵. Second, each IV was also systematically queried against the GWAS Catalog (https://www.ebi.ac.uk/gwas/) to identify any previously reported associations with the outcome or its major risk factors using the same stringent threshold. Any IV demonstrating a significant association with these traits was presumed to exhibit horizontal pleiotropy and was consequently removed from the final set of IVs used in our analysis ***(S3 and S4 Table)***.” |
|  | d) | For each exposure, outcome, and other relevant variables, describe methods of assessment and diagnostic criteria for diseases | 39 | “Causal effects were estimated for both directions primarily using the inverse variance weighted (IVW) method; a random-effects model was employed in the presence of significant heterogeneity, otherwise, a fixed-effect model was used (70, 71). And this was supplemented by weighted median, MR-Egger, simple mode, and weighted mode approaches for robustness and sensitivity (20). Heterogeneity among IVs was assessed via Cochran’s Q test (*P* < 0.05 indicating significant heterogeneity) and quantified using the I² statistic. Furthermore, the MR-PRESSO method was used to detect and correct for potential horizontal pleiotropic outliers (72). The leave-one-out sensitivity analysis was performed to evaluate the influence of individual SNPs on the results (20). Finally, a meta-analysis was performed to synthesize the causal estimates from the discovery and replication cohorts to derive a summary effect (73, 74).” |
|  | e) | Provide details of ethics committee approval and participant informed consent, if relevant |  | Not Applicate |
| 5 | **Assumptions** | Explicitly state the three core IV assumptions for the main analysis (relevance, independence and exclusion restriction) as well assumptions for any additional or sensitivity analysis | 37-38 | “The MR analysis relied on three core assumptions: the relevance assumption requiring a strong association between instrumental variable (IV) and exposure; the independence assumption ensuring IVs are unaffected by confounders; and the exclusion restriction assumption stipulating that IVs influence the outcome solely through the exposure.” |
| 6 | **Statistical methods: main analysis** | Describe statistical methods and statistics used |  |  |
|  | a) | Describe how quantitative variables were handled in the analyses (i.e., scale, units, model) |  | Not Applicate. The harmonized GWAS summary statistics we used were from other studies. |
|  | b) | Describe how genetic variants were handled in the analyses and, if applicable, how their weights were selected |  | Not Applicate. The harmonized GWAS summary statistics we used were from other studies. |
|  | c) | Describe the MR estimator (e.g. two-stage least squares, Wald ratio) and related statistics. Detail the included covariates and, in case of two-sample MR, whether the same covariate set was used for adjustment in the two samples | 37-39 | Our study primarily employed a Bidirectional Mendelian Randomization design, utilizing publicly available Genome-Wide Association Study (GWAS) summary statistics. Causal effects were estimated using the inverse variance weighted (IVW) method as the primary approach , supplemented by the weighted median, MR-Egger, simple mode, and weighted mode methods for robustness checks. Related statistical analyses included Cochran’s Q test to assess heterogeneity among instrumental variables, MR-PRESSO method to detect and correct for potential horizontal pleiotropic outliers and the leave-one-out sensitivity analysis to evaluate the influence of individual SNPs. Regarding covariate adjustment, as we used summary-level data, adjustments for covariates such as age, sex, and population structure via principal components were assumed to have been performed by the consortia that conducted the original GWAS. |
|  | d) | Explain how missing data were addressed |  | Not Applicate. The harmonized GWAS summary statistics we used were from other studies. |
|  | e) | If applicable, indicate how multiple testing was addressed | 37 | This study employed a bidirectional MR approach to explore the causal relationship between hypothyroidism and cirrhosis. The primary analyses was conducted using the discovery cohorts and subsequently performed in the various validation cohorts for both the forward (hypothyroidism to cirrhosis) and reverse (cirrhosis to hypothyroidism) directions. |
| 7 | **Assessment of assumptions** | Describe any methods or prior knowledge used to assess the assumptions or justify their validity | 37-39 | In our study, we employed several methods to rigorously assess the validity of the three core Mendelian Randomization (MR) assumptions. To satisfy the relevance assumption, Genetic instruments for each direction were selected based on genome-wide significance (*P* < 5 × 10⁻⁸) and refined using LD clumping thresholds (*R*² = 0.001, clumping distance = 10,000 kb) to ensure independence. Single Nucleotide Polymorphisms (SNPs) with F-statistics < 10, calculated as *F* = $\frac{R^{2}\times(N-1-k)}{(1-R^{2})\times k}$ where 𝑅^2^ is the variance explained by IVs, *N* is the sample size, and *k* is the number of IVs, were excluded to minimize weak instrument bias (68). To further ensure the correct causal direction, we applied Steiger filtering, removing any IVs that explained more variance in the outcome than in the exposure (69). To rigorously uphold the core assumptions of MR, we coned a comprehensive screening for potential horizontal pleiotropy. First, we directly screened all IVs against the outcome and removed any variants associated with it that surpassed a significance threshold of *P* < 1 × 10⁻⁵. Second, each IV was also systematically queried against the GWAS Catalog (https://www.ebi.ac.uk/gwas/) to identify any previously reported associations with the outcome or its major risk factors using the same stringent threshold. Any IV demonstrating a significant association with these traits was presumed to exhibit horizontal pleiotropy and was consequently removed from the final set of IVs used in our analysis ***(S3 and S4 Table)***.  Causal effects were estimated for both directions primarily using the inverse variance weighted (IVW) method; a random-effects model was employed in the presence of significant heterogeneity, otherwise, a fixed-effect model was used (70, 71). And this was supplemented by weighted median, MR-Egger, simple mode, and weighted mode approaches for robustness and sensitivity (20). Heterogeneity among IVs was assessed via Cochran’s Q test (*P* < 0.05 indicating significant heterogeneity) and quantified using the I² statistic. Furthermore, the MR-PRESSO method was used to detect and correct for potential horizontal pleiotropic outliers (72). The leave-one-out sensitivity analysis was performed to evaluate the influence of individual SNPs on the results (20). Finally, a meta-analysis was performed to synthesize the causal estimates from the discovery and replication cohorts to derive a summary effect (73, 74). |
| 8 | **Sensitivity analyses and additional analyses** | Describe any sensitivity analyses or additional analyses performed (e.g. comparison of effect estimates from different approaches, independent replication, bias analytic techniques, validation of instruments, simulations) | 38-39 | Genetic instruments for each direction were selected based on genome-wide significance (*P* < 5 × 10⁻⁸) and refined using LD clumping thresholds (*R*² = 0.001, clumping distance = 10,000 kb) to ensure independence. Single Nucleotide Polymorphisms (SNPs) with F-statistics < 10, calculated as *F* = $\frac{R^{2}\times(N-1-k)}{(1-R^{2})\times k}$ where 𝑅^2^ is the variance explained by IVs, *N* is the sample size, and *k* is the number of IVs, were excluded to minimize weak instrument bias (68). To further ensure the correct causal direction, we applied Steiger filtering, removing any IVs that explained more variance in the outcome than in the exposure (69). To rigorously uphold the core assumptions of MR, we coned a comprehensive screening for potential horizontal pleiotropy. First, we directly screened all IVs against the outcome and removed any variants associated with it that surpassed a significance threshold of *P* < 1 × 10⁻⁵. Second, each IV was also systematically queried against the GWAS Catalog (https://www.ebi.ac.uk/gwas/) to identify any previously reported associations with the outcome or its major risk factors using the same stringent threshold. Any IV demonstrating a significant association with these traits was presumed to exhibit horizontal pleiotropy and was consequently removed from the final set of IVs used in our analysis ***(S3 and S4 Table)***.  Causal effects were estimated for both directions primarily using the inverse variance weighted (IVW) method; a random-effects model was employed in the presence of significant heterogeneity, otherwise, a fixed-effect model was used (70, 71). And this was supplemented by weighted median, MR-Egger, simple mode, and weighted mode approaches for robustness and sensitivity (20). Heterogeneity among IVs was assessed via Cochran’s Q test (*P* < 0.05 indicating significant heterogeneity) and quantified using the I² statistic. Furthermore, the MR-PRESSO method was used to detect and correct for potential horizontal pleiotropic outliers (72). The leave-one-out sensitivity analysis was performed to evaluate the influence of individual SNPs on the results (20). Finally, a meta-analysis was performed to synthesize the causal estimates from the discovery and replication cohorts to derive a summary effect (73, 74). |
| 9 | **Software and pre-registration** |  |  |  |
|  | a) | Name statistical software and package(s), including version and settings used |  | The MR analysis utilizes the R language and the Python programming software. The core analyses were performed using the 'TwoSampleMR' R package and its associated dependencies to execute the Bidirectional MR calculations and subsequent sensitivity analyses. |
|  | b) | State whether the study protocol and details were pre-registered (as well as when and where) |  | Not Applicate |
|  | **RESULTS** |  |  |  |
| 10 | **Descriptive data** |  |  |  |
|  | a) | Report the numbers of individuals at each stage of included studies and reasons for exclusion. Consider use of a flow diagram |  | Not Applicate. The harmonized GWAS summary statistics we used were from other studies. |
|  | b) | Report summary statistics for phenotypic exposure(s), outcome(s), and other relevant variables (e.g. means, SDs, proportions) |  | Not Applicate. The harmonized GWAS summary statistics we used were from other studies. |
|  | c) | If the data sources include meta-analyses of previous studies, provide the assessments of heterogeneity across these studies |  | Not Applicate. The harmonized GWAS summary statistics we used were from other studies. |
|  | d) | For two-sample MR:  i.  Provide justification of the similarity of the genetic variant-exposure associations between the exposure and outcome samples  ii.  Provide information on the number of individuals who overlap between the exposure and outcome studies |  | Not Applicate. The harmonized GWAS summary statistics we used were from other studies. |
| 11 | **Main results** |  |  |  |
|  | a) | Report the associations between genetic variant and exposure, and between genetic variant and outcome, preferably on an interpretable scale |  | Not Applicate. |
|  | b) | Report MR estimates of the relationship between exposure and outcome, and the measures of uncertainty from the MR analysis, on an interpretable scale, such as odds ratio or relative risk per SD difference | 14-15 | “The primary forward MR analysis, using the discovery cohorts for both the exposure and outcome, demonstrated a significant causal association between hypothyroidism and an increased risk of cirrhosis in fixed model (*P* = 9.7 × 10^-4^, OR [95% CI] = 1.06 [1.02–1.09]). This analysis showed no evidence of both directional horizontal pleiotropy (*P* = 0.81) and heterogeneity (*P* = 0.35). To rigorously assess the robustness of this finding, we conducted three additional analyses using different combinations of discovery and replication datasets. These analyses largely supported our primary conclusion, showing a consistent direction of effect. Specifically, two of them also yielded statistically significant associations (*P* = 3.6 × 10^-3^, OR [95% CI] = 1.06 [1.02–1.09]; *P* = 8.4 × 10^-3^, OR [95% CI] = 1.08 [1.02–1.15]). Although one of the finding, utilizing the datasets both from Finngen consortium, did not reach statistical significance (*P* = 8.4 × 10^-2^, OR [95% CI] = 1.05 [0.99–1.11]), the effect was directionally consistent with our primary result. Crucially, to synthesize the evidence across all four findings, a meta-analysis was conducted. This provided a robust summary estimate, yielding strong and consistent evidence for a causal link between hypothyroidism and an increased risk of cirrhosis (*P* < 1 × 10^-4^, OR [95% CI] = 1.06 [1.04–1.08]). “Detailed results for all MR analyses and additional statistics were presented in **S5 Table**. |
|  | c) | If relevant, consider translating estimates of relative risk into absolute risk for a meaningful time period |  | Not Applicate |
|  | d) | Consider plots to visualize results (e.g. forest plot, scatterplot of associations between genetic variants and outcome versus between genetic variants and exposure) |  | **Figure 3A** |
| 12 | **Assessment of assumptions** |  |  |  |
|  | a) | Report the assessment of the validity of the assumptions | 14-15 | “The primary forward MR analysis, using the discovery cohorts for both the exposure and outcome, demonstrated a significant causal association between hypothyroidism and an increased risk of cirrhosis in fixed model (*P* = 9.7 × 10^-4^, OR [95% CI] = 1.06 [1.02–1.09]). This analysis showed no evidence of both directional horizontal pleiotropy (*P* = 0.81) and heterogeneity (*P* = 0.35). To rigorously assess the robustness of this finding, we conducted three additional analyses using different combinations of discovery and replication datasets. These analyses largely supported our primary conclusion, showing a consistent direction of effect. Specifically, two of them also yielded statistically significant associations (*P* = 3.6 × 10^-3^, OR [95% CI] = 1.06 [1.02–1.09]; *P* = 8.4 × 10^-3^, OR [95% CI] = 1.08 [1.02–1.15]). Although one of the finding, utilizing the datasets both from Finngen consortium, did not reach statistical significance (*P* = 8.4 × 10^-2^, OR [95% CI] = 1.05 [0.99–1.11]), the effect was directionally consistent with our primary result. Crucially, to synthesize the evidence across all four findings, a meta-analysis was conducted. This provided a robust summary estimate, yielding strong and consistent evidence for a causal link between hypothyroidism and an increased risk of cirrhosis (*P* < 1 × 10^-4^, OR [95% CI] = 1.06 [1.04–1.08]).  In contrast, the reverse MR analysis revealed no evidence of a causal effect of cirrhosis on hypothyroidism. The results were consistently non-significant across all four analytical combinations.”Detailed results for all MR analyses and additional statistics were presented in **S5 Table**. |
|  | b) | Report any additional statistics (e.g., assessments of heterogeneity across genetic variants, such as *I^2^*, Q statistic or E-value) | 14-15 | Detailed results for all MR analyses and additional statistics were presented in **S5 Table**. |
| 13 | **Sensitivity analyses and additional analyses** |  |  |  |
|  | a) | Report any sensitivity analyses to assess the robustness of the main results to violations of the assumptions | 14-15 | “The primary forward MR analysis, using the discovery cohorts for both the exposure and outcome, demonstrated a significant causal association between hypothyroidism and an increased risk of cirrhosis (OR = 1.08, 95% CI: 1.04–1.12; P = 6.7 × 10⁻⁵). This analysis showed no evidence of directional horizontal pleiotropy (P = 0.66), although modest heterogeneity was noted (P = 0.04). Crucially, this causal association was successfully replicated using the independent validation cohorts, yielding a consistent and significant casual effect (OR = 1.06, 95% CI: 1.01-1.12; P = 0.032), with no significant pleiotropy (P = 0.56) detected. Furthermore, the effect estimates remained robust in additional sensitivity analyses combining discovery and validation datasets **(S4 Table)**. Collectively, these findings provide strong and consistent evidence for a causal link between hypothyroidism and an increased risk of cirrhosis.  In contrast, the reverse MR analysis revealed no evidence of a causal effect of cirrhosis on hypothyroidism. The results were consistently non-significant across all four analytical combinations **(S5 Table, Fig 3A)**.“ |
|  | b) | Report results from other sensitivity analyses or additional analyses | 14-15 | Detailed results for all MR analyses and additional statistics were presented in **S5 Table**. |
|  | c) | Report any assessment of direction of causal relationship (e.g., bidirectional MR) | 14-15 | “The primary forward MR analysis, using the discovery cohorts for both the exposure and outcome, demonstrated a significant causal association between hypothyroidism and an increased risk of cirrhosis in fixed model (*P* = 9.7 × 10^-4^, OR [95% CI] = 1.06 [1.02–1.09]). This analysis showed no evidence of both directional horizontal pleiotropy (*P* = 0.81) and heterogeneity (*P* = 0.35). To rigorously assess the robustness of this finding, we conducted three additional analyses using different combinations of discovery and replication datasets. These analyses largely supported our primary conclusion, showing a consistent direction of effect. Specifically, two of them also yielded statistically significant associations (*P* = 3.6 × 10^-3^, OR [95% CI] = 1.06 [1.02–1.09]; *P* = 8.4 × 10^-3^, OR [95% CI] = 1.08 [1.02–1.15]). Although one of the finding, utilizing the datasets both from Finngen consortium, did not reach statistical significance (*P* = 8.4 × 10^-2^, OR [95% CI] = 1.05 [0.99–1.11]), the effect was directionally consistent with our primary result. Crucially, to synthesize the evidence across all four findings, a meta-analysis was conducted. This provided a robust summary estimate, yielding strong and consistent evidence for a causal link between hypothyroidism and an increased risk of cirrhosis (*P* < 1 × 10^-4^, OR [95% CI] = 1.06 [1.04–1.08]).  In contrast, the reverse MR analysis revealed no evidence of a causal effect of cirrhosis on hypothyroidism. The results were consistently non-significant across all four analytical combinations ***(S5 Table, Fig 3A)***.“ |
|  | d) | When relevant, report and compare with estimates from non-MR analyses |  | Not Applicate |
|  | e) | Consider additional plots to visualize results (e.g., leave-one-out analyses) |  | Not Applicate |
|  | **DISCUSSION** |  |  |  |
| 14 | **Key results** | Summarize key results with reference to study objectives | 24 | “This study provides the first insight into the causal associations, potential biomarkers, and drug targets between hypothyroidism and cirrhosis through clinical cohort and integrated multi-omics approaches.“ |
| 15 | **Limitations** | Discuss limitations of the study, taking into account the validity of the IV assumptions, other sources of potential bias, and imprecision. Discuss both direction and magnitude of any potential bias and any efforts to address them | 32-33 | “Despite yielding several important findings, our study is not without limitations. First, our research was primarily based on European population data, which may limit the generalizability of the results to other ethnic groups. that hypothyroidism and cirrhosis may have different disease characteristics and risk factors in Asian populations, future validation in diverse populations is needed. Second, because the control group was less likely than the case group to undergo relevant clinical examinations during treatment, our retrospective analysis resulted in substantial missing data. This differential missingness may have, in turn, introduced selection bias; consequently, our observational findings warrant validation in future prospective studies. Third, in the transcriptome data analysis, owing to the lack of bulk RNA-seq data for hypothyroidism, we used HT data as a substitute. Although HT is one of the most common causes of hypothyroidism, its distinct immunological characteristics might lead to an overemphasis on autoimmune-specific pathways, potentially conflating them with the features of hypothyroidism itself. Therefore, future studies on transcriptomic data from patients with well-defined hypothyroidism are necessary to validate our findings. Finally, our study primarily focused on the immunological mechanisms of the hypothyroidism-cirrhosis relationship, while other potential mechanisms, such as metabolic disorders and hormonal influences, require further investigation. Considering the important role of thyroid hormones in liver metabolism, integrating metabolomics data and thyroid hormone signaling pathway analysis may provide additional insights into disease mechanisms“ |
| 16 | **Interpretation** |  |  |  |
|  | a) | Meaning: Give a cautious overall interpretation of results in the context of their limitations and in comparison with other studies | 24，26 | “This study provides the first insight into the causal associations, potential biomarkers, and drug targets between hypothyroidism and cirrhosis through clinical cohort and integrated multi-omics approaches.“  “More importantly, it revealed a unidirectional causal effect of hypothyroidism on the risk of developing cirrhosis “ |
|  | b) | Mechanism: Discuss underlying biological mechanisms that could drive a potential causal relationship between the investigated exposure and the outcome, and whether the gene-environment equivalence assumption is reasonable. Use causal language carefully, clarifying that IV estimates may provide causal effects only under certain assumptions | 26-27 | “To progress from genetic association to causal inference, MR was applied. More importantly, it revealed a unidirectional causal effect of hypothyroidism on the risk of developing cirrhosis, with the finding being robust across both discovery and replication cohorts and showing no evidence of horizontal pleiotropy. This causal inference implies that hypothyroidism may actively contribute to the of cirrhosis, rather than merely representing a secondary manifestation of impaired liver function. Supporting this, previous studies have demonstrated that functional genes mediating the effects of thyroid hormones, which are essential for regulating fatty acid β-oxidation, also participate in hepatic lipid metabolism (38, 39). Furthermore, in hypothyroid states, reduced T3 levels suppress fatty acid oxidation and cholesterol clearance pathways in hepatocytes, leading to lipid accumulation. Such metabolic dysregulation contributes to the onset of hepatic steatosis and its progression toward fibrosis (40, 41). These findings not only reinforce our research’s results but also highlight the potential for clinical translation. By identifying hypothyroidism as an upstream risk factor in the pathogenesis of cirrhosis, our results suggest that early control of thyroid dysfunction may provide a novel avenue for the prevention and treatment of liver cirrhosis.“ |
|  | c) | Clinical relevance: Discuss whether the results have clinical or public policy relevance, and to what extent they inform effect sizes of possible interventions | 24-25 | “This study provides the first insight into the causal associations, potential biomarkers, and drug targets between hypothyroidism and cirrhosis through clinical cohort and integrated multi-omics approaches. We initially identified a significantly higher prevalence of both overt and subclinical hypothyroidism among cirrhotic patients compared to healthy controls in a large clinical cohort. This association remained significant even after adjusting for multiple covariates, suggesting that hypothyroidism is an independent risk factor for cirrhosis. Chronic hepatic inflammation and activation of hepatic stellate cells (HSCs) are key events in the pathogenesis of cirrhosis. One of the potential core driver of these processes is the strong association between hypothyroidism and insulin resistance. Thyroid hormone imbalance impairs mitochondrial function and energy metabolism, thereby exacerbating insulin resistance (28, 29). Under insulin-resistant conditions, Kupffer cells release pro-inflammatory cytokines such as Tumor necrosis factor α and Interleukin 6, which subsequently activate HSCs to produce excessive collagen and other extracellular matrix components, accelerating hepatic fibrogenesis and ultimately leading to cirrhosis (30, 31). In addition, hypothyroidism can disrupt the gut microbiota composition and impair intestinal barrier integrity, reduce gastrointestinal motility, and alter luminal potential of hydrogen (pH). These disturbances increase intestinal permeability and facilitate the translocation of bacterial endotoxins into the liver via the portal vein. This process activates Toll-like receptor 4 and upregulates the NF-κB signaling pathway, further promoting the risk of cirrhosis (32-34).“ |
| 17 | **Generalizability** | Discuss the generalizability of the study results (a) to other populations, (b) across other exposure periods/timings, and (c) across other levels of exposure | 32 | “Despite yielding several important findings, our study is not without limitations. First, our research was primarily based on European population data, which may limit the generalizability of the results to other ethnic groups. that hypothyroidism and cirrhosis may have different disease characteristics and risk factors in Asian populations, future validation in diverse populations is needed.” |
|  | **OTHER INFORMATION** |  |  |  |
| 18 | **Funding** | Describe sources of funding and the role of funders in the present study and, if applicable, sources of funding for the databases and original study or studies on which the present study is based | 64 | “This work was supported by the Guangdong Basic and Applied Basic Research Foundation (Grant Number 2019A1515110060 to Jiyuan Zhou), the Guangzhou Science and Technology Department-School Joint Project (grant number 2023A03J0417 to Jiyuan Zhou), and the Plan on Enhancing Scientific Research in GMU (Grant Number 02-410-2302035XM to Jiyuan Zhou). The funders had no role in study design, data collection and analysis, decision to publish, or preparation of the manuscript.“ |
| 19 | **Data and data sharing** | Provide the data used to perform all analyses or report where and how the data can be accessed, and reference these sources in the article. Provide the statistical code needed to reproduce the results in the article, or report whether the code is publicly accessible and if so, where | 63 | “This work was supported by the Guangdong Basic and Applied Basic Research Foundation (Grant Number 2019A1515110060 to Jiyuan Zhou), the Guangzhou Science and Technology Department-School Joint Project (grant number 2023A03J0417 to Jiyuan Zhou), and the Plan on Enhancing Scientific Research in GMU (Grant Number 02-410-2302035XM to Jiyuan Zhou). The funders had no role in study design, data collection and analysis, decision to publish, or preparation of the manuscript.“ |
| 20 | **Conflicts of Interest** | All authors should declare all potential conflicts of interest |  | The authors declare that there is no conflict of interest. |

This checklist is copyrighted by the Equator Network under the Creative Commons Attribution 3.0 Unported (CC BY 3.0) license.

1. Skrivankova VW, Richmond RC, Woolf BAR, Yarmolinsky J, Davies NM, Swanson SA, et al. Strengthening the Reporting of Observational Studies in Epidemiology using Mendelian Randomization (STROBE-MR) Statement. JAMA. 2021;under review.

2. Skrivankova VW, Richmond RC, Woolf BAR, Davies NM, Swanson SA, VanderWeele TJ, et al. Strengthening the Reporting of Observational Studies in Epidemiology using Mendelian Randomisation (STROBE-MR): Explanation and Elaboration. BMJ. 2021;375:n2233.
