# Supplementary figures and images for: The role of hypothyroidism in cirrhosis pathogenesis: A retrospective cohort study and multi-omics integration analysis
Source: PLoS Genet. 2025 Nov 7;21(11):e1011947. doi: 10.1371/journal.pgen.1011947 (PMC12611128; doi:10.1371/journal.pgen.1011947)

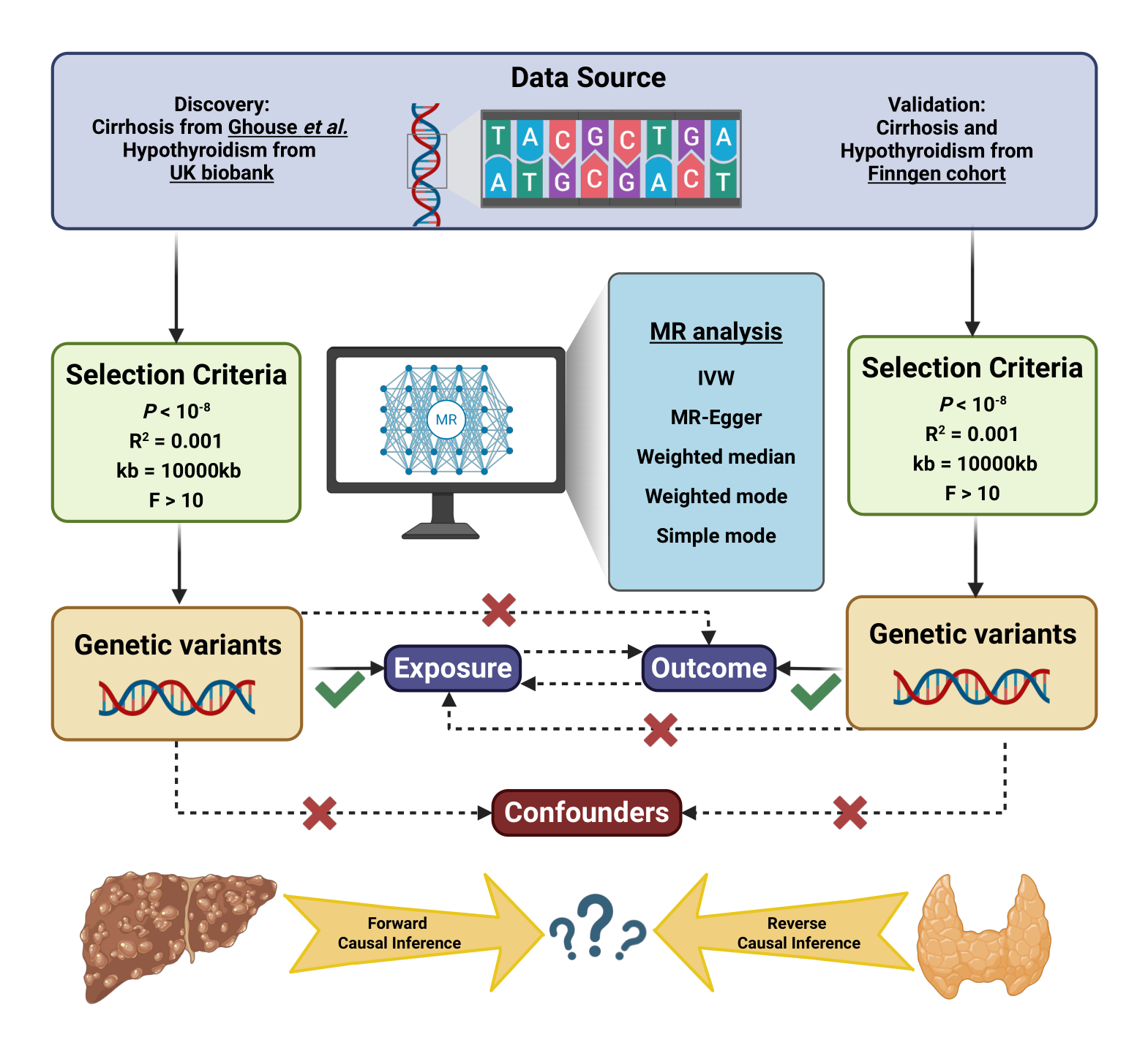

Supplement: S1 Fig — https://BioRender.com/484biwe). (TIFF) [file pgen.1011947.s017.tiff]
