# Supplementary material for: The role of hypothyroidism in cirrhosis pathogenesis: A retrospective cohort study and multi-omics integration analysis
Source: PLoS Genet. 2025 Nov 7;21(11):e1011947. doi: 10.1371/journal.pgen.1011947 (PMC12611128; doi:10.1371/journal.pgen.1011947)
Supplement: S1 Text — (DOCX) [file pgen.1011947.s018.docx]

**Supplementary Methods**

**Weighted Gene Co-expression Network Analysis (WGCNA)**

To validate the biological pathways shared between hypothyroidism and cirrhosis, we employed WGCNA, a systems biology method for identifying modules of highly co-expressed genes that are associated with clinical traits (1).

The analysis was initiated with the transcriptomic datasets from both hypothyroidism and cirrhosis. A critical preprocessing step involved filtering the genes to enhance the signal-to-noise ratio. This approach prioritizes genes with dynamic expression profiles, which are more likely to be biologically informative.

A signed co-expression network was then constructed based on pairwise Pearson correlations. To approximate a scale-free topology, a fundamental property of many biological networks, a soft-thresholding power (β) was selected. This is achieved by raising the absolute value of the correlation to the power of β, a process that emphasizes strong correlations and effectively suppresses weak ones, thus reducing the noise from trivial correlations. The resulting adjacency matrix was subsequently transformed into a Topological Overlap Matrix (TOM). The TOM provides a more robust and biologically meaningful measure of gene interconnectedness by considering not only the direct correlation between two genes but also the extent to which they share connections with other genes in the network.

Using the TOM-based dissimilarity measure, genes were subjected to average linkage hierarchical clustering. A "gene module" was then defined as a cluster of densely interconnected genes, formally identified by applying the dynamic tree-cutting algorithm to the clustering dendrogram. Each module, representing a group of genes with coordinated expression patterns that likely reflect shared biological functions or regulatory pathways, was assigned a unique color for identification.

Finally, to relate these modules to the clinical phenotypes, the module eigengene (ME) was calculated for each module. The ME, defined as the first principal component of the module's expression matrix, serves as a single, representative expression profile for all genes within that module. We then correlated each ME with the clinical trait of interest.

**Statistical Model for Estimating the Overall Genetic Effect**

To conduct a comprehensive analysis of the overall effect for each gene on 675 disease traits, we utilized a random-effects meta-analysis framework (2). For each gene, we estimated the overall log odds ratio (logOR) of gene expression on disease outcomes across 675 diseases, accounting for the variability in effect sizes across diseases. The random-effects model for estimating the overall effect size of each gene is given by:

$$\beta_{i}\mid\theta_{i}\sim\mathcal{N}\left( \theta_{i},SE_{i}^{2} \right)$$

$$\theta_{i}\sim\mathcal{N}\left( \mu_{g},\tau^{2} \right)$$

This two-stage hierarchical model first specified that the observed effect size for disease ($\beta_{i}$) was drawn from a normal distribution centered on the true effect ($\theta_{i}$) with a known variance ($\mathrm{SE}_{i}^{2}$). In the second stage, $\theta_{i}$ were treated as random variables, assumed to follow a common distribution for gene with a mean ($\mu_{g}$) and variance ($\tau^{2}$). Within this framework, $\mu_{g}$ represented the overall summary effect for the gene across all diseases, while $\tau^{2}$ quantified the magnitude of between-disease heterogeneity.

The overall effect size for each gene was estimated by weighting the individual effect sizes using the inverse of their variances, as follows:

$$\hat{\mu_{g}}=\frac{\sum_{i=1}^{m_{g}} \frac{\beta_{i}}{SE_{i}^{2}+\hat{\tau^{2}}}}{\sum_{i=1}^{m_{g}} \frac{1}{SE_{i}^{2}+\hat{\tau^{2}}}}$$

In this calculation, the overall effect $\hat{\mu_{g}}$ act as a weighted average of the individual effect sizes ($\beta_{i}$). The weight assigned to each effect was the inverse of a pooled variance term. This term summed the variance of the individual effect estimate ($\mathrm{SE}_{i}^{2}$) with the estimated random-effects variance ($\tau^{2}$), the latter of which accounted for the heterogeneity observed across all diseases.

The overall OR for each gene was then calculated by exponentiating the overall log odds ratio $\hat{\mu_{g}}$:

$$\hat{\text{OR}_{g}}=\exp\left( \hat{\mu_{g}} \right)$$

The aggregate effect of each gene was categorized based on the direction and statistical significance of this OR. The effect was classified as harmful for an $\hat{\text{OR}_{g}}$> 1 with P*_overall_* < 0.05, protective for an $\hat{\text{OR}_{g}}$< 1 with P*_overall_* < 0.05, and otherwise considered non-significant.

References

1. Langfelder P, Horvath S. WGCNA: an R package for weighted correlation network analysis. BMC Bioinformatics. 2008;9:559.

2. Zhou W, Nielsen JB, Fritsche LG, Dey R, Gabrielsen ME, Wolford BN, et al. Efficiently controlling for case-control imbalance and sample relatedness in large-scale genetic association studies. Nat Genet. 2018;50(9):1335–41.
