# Supplementary material for: The role of hypothyroidism in cirrhosis pathogenesis: A retrospective cohort study and multi-omics integration analysis
Source: PLoS Genet. 2025 Nov 7;21(11):e1011947. doi: 10.1371/journal.pgen.1011947 (PMC12611128; doi:10.1371/journal.pgen.1011947)
Supplement: Attachment — Submitted filename: Response to Reviewers.docx [file pgen.1011947.s020.docx]

**Response to Reviewer 1：**

Dr Zhou and colleagues investigated the genetic overlap between hypothyroidism and liver cirrhosis using publicly available genome-wide association study (GWAS) summary statistics from large biobanks such as UK Biobank and FinnGen. The authors report a significant observational association between the two diseases based on MIMIC-IV data, a significant genetic correlation between published GWAS of the two diseases using LD score regression, and a significant causal effect of hypothyroidism on cirrhosis risk (but no evidence of a causal effect of cirrhosis on hypothyroidism risk) which was replicated in an independent cohort. The authors then incorporated genomic data from multiple databases which highlighted HLA-DQA1 and CD27 as key molecules linking hypothyroidism and liver cirrhosis.

Thank you to the authors for sharing this interesting study. Overall, I think the manuscript is clear and well written. I have no major concerns at this time. However, I invite the authors to address the following minor comments/suggestions to improve clarity:

**Response:** Thank you for your positive feedback and for acknowledging the clarity of our manuscript. We appreciate the opportunity to address the following minor comments and suggestions to further improve the manuscript.

1. Line 151-153: A lot of individuals were excluded from the retrospective analysis due to missing data (~36% of cirrhosis cases and ~66% of cirrhosis controls excluded). What are the reasons for this level of missing data? Please discuss whether differences in the missingness between cases and controls could have given rise to the observational associations reported.

**Response:** Thank you for raising this point. Our retrospective cohort is drawn from MIMIC-IV, where variables are recorded as part of routine clinical care rather than by a standardized research protocol. Missingness primarily reflects: (i) inconsistent documentation of lifestyle and anthropometric measures (e.g., smoking status, BMI); (ii) limited ordering/availability of certain laboratory tests within the analytic window; and (iii) inpatient workflow differences whereby putative controls were less likely than cirrhosis cases to undergo liver-related testing, resulting in a higher missingness rate among controls. We now present an inclusion–exclusion flow diagram and a baseline characteristics table in the Results. We have also expanded the Discussion to describe the missing-data mechanism and to note the potential for selection bias arising from differential missingness between cases and controls, which we explicitly acknowledge as a study limitation.

**Modification to the Manuscript:** The details can be seen in lines 604-609, page 31-32.

2. Line 159-165: Are there any other important covariates that may act as confounders for these associations – smoking, BMI, medication?

**Response:** Thank you for pointing out the potential confounders of smoking, BMI, and medication use. In MIMIC-IV, medication records are highly heterogeneous across units and hospitalization stages (e.g., frequent omissions, inconsistent timestamps, and non-standardized indication/ordering fields), which prevents construction of a reliable, comparable exposure measure; therefore, medications were not included as covariates, which helps avoid introducing non-comparable or misclassified exposure variables that could bias the estimates. The database likewise lacks clear, standardized indicators for smoking and alcohol use. For BMI, we derived weight status and adjusted for it in multivariable models; our main findings remained statistically significant after this adjustment. We describe covariate selection and handling in the Methods and Results, and we acknowledge in the Discussion the possibility of residual confounding and its implications for interpretation.

**Modification to the Manuscript:** The details can be seen in lines 169-178, page 9; lines 604-609, page 31-32; lines 684-687, page 35 and Table1.

3. Line 185-187: The genetic correlation is technically statistically significant, but the effect estimate is fairly weak (rg = 0.1566). I would suggest adding some text to the discussion to highlight the weak magnitude of effect.

**Response:** We sincerely appreciate for this insightful comment and for highlighting the crucial point about interpreting the magnitude of the genetic correlation. We would like to clarify that the value you cited (rg = 0.1566) was from a preliminary analysis. In our revised manuscript, we have updated our methodology to be more robust. Specifically, as described in the Methods section ("Genetic correlation analysis"), we have converted the SNP-based heritability from the observed scale to the more appropriate liability scale for both hypothyroidism and cirrhosis.

This corrected analysis yields a revised genetic correlation of rg = 0.2135 (P = 4 × 10⁻⁴), which is now accurately reported in the Results section. Furthermore, we completely agree with your excellent suggestion that the magnitude of this effect warrants careful discussion. Although the updated correlation value is slightly higher, it still represents a modest, rather than a strong, shared genetic architecture. To address this point directly, we have now added a paragraph to the Discussion section to elaborate on this finding.

**Modification to the Manuscript:** The details can be seen in line 198, page 13; lines 450-468, page 24-25 and lines 700-710, page 36.

4. Line 187-190: Can you comment on how these SNP heritabilities compare to estimates from twin studies for hypothyroidism and liver cirrhosis (where possible)? Is it accurate to describe a SNP heritability of 4.56% as indicating genetic factors play a “prominent role” in hypothyroidism? Are you able to make comparisons to SNP heritabilities based on common variant GWAS for other relevant disease traits?

**Response:** Thank you for this important point, which prompted us to re-evaluate our heritability reporting. We agree that the original SNP-heritability estimate of 4.56% on the observed scale does not justify describing genetic effects as “prominent.” In the revision, we convert SNP-heritability from observed scale to the liability scale, which is the standard for case–control traits and provides a more accurate reflection of population risk. Using prevalence assumptions from the literature and GBD, the liability-scale estimates are 17.64% (SE 1.48%) for hypothyroidism and 12.93% (SE 2.10%) for cirrhosis. We have therefore revised the wording from “prominent” to “substantial” to more accurately reflect these magnitudes.

It is well established that SNP-based heritability, which captures the contribution of common variants measured in GWAS, is lower than the total heritability estimated from twin studies, which also incorporate rare and structural variants (1). For hypothyroidism, which is mainly caused by autoimmune thyroid disease, twin studies have reported heritability estimates as high as 55% (2). Our liability-scale estimate of 17.64% is consistent with this expectation and represents the common-variant component. For cirrhosis, direct twin data are scarce, but its main precursor hepatic fibrosis has an estimated heritability of about 50% (3). Our estimate of 12.93% therefore indicates a meaningful contribution of common variants to the risk of progression.

To provide broader context, liability-scale SNP-heritability estimates for other complex diseases often fall in a similar range, such as asthma (15%), hypertension (25%), dyslipidemia (26%), and type 2 diabetes (30%) (4). Our revised estimates for hypothyroidism and cirrhosis are therefore comparable to these values and support the conclusion that genetic factors make a substantial contribution to both conditions.

**Modification to the Manuscript:** The details can be seen in lines 199-204, page 13 and lines 700-710, page 36.

5. Line 200: Is it correct to refer to this as “progression of cirrhosis”? Line 208 instead refers to the outcome as cirrhosis risk. If I understood correctly, it’s later in the paper you include hepatitis as an intermediate progression trait.

**Response:** Thank you for pointing out the inconsistency in terminology. We agree that the term "progression of cirrhosis" should be unified with the phrase "cirrhosis risk" to maintain clarity and consistency throughout the manuscript. As suggested, we will revise the manuscript to consistently refer to the outcome as "cirrhosis risk" rather than "progression of cirrhosis."

**Modification to the Manuscript:** The relevant expressions in the text have been revised.

6. Line 240-243: It’s not clear to me what is meant by a “gene module”. I think this requires further explanation before discussing the results from WGCNA. In addition, I suggest adding some context for whether a network of 4393 genes for the hypothyroidism analysis is meaningful given that this would represent a significant proportion of all genes in the human genome. Similarly for cirrhosis. In particular, is it surprising to find significant phenotypic correlation given the number of genes reported?

**Response:** Thank you for your comment. To clarify, in Weighted Gene Co-expression Network Analysis (WGCNA), a "gene module" refers to a group of co-expressed genes involved in similar biological processes. The large network of 4393 genes in our hypothyroidism analysis reflects the complexity of the disease's biological mechanisms. While this network is extensive, it remains meaningful as it represents coordinated gene expression patterns rather than individual gene significance. The observed phenotypic correlation is consistent with the co-regulated nature of these genes. We have now provided a more detailed explanation of this methodology in the "Supplementary Methods" section, including how gene modules were identified and their relevance to hypothyroidism and cirrhosis.

**Modification to the Manuscript:** The details can be seen in lines 780-782, page 40 and Supplementary Methods.

7. General: How sensitive are the multi-omics approaches to the GWAS summary statistics used? Given that this was based on common variant GWAS, how much would be missing by not including rare variants and more complex genetic variation such as structural variants in the input to these methods?

**Response:** Thank you for raising the issues of data sensitivity and variant scope. To address sensitivity to any single GWAS and to ensure robustness, we used a two-stage discovery-and-replication design for all causal-inference analyses. In discovery, we selected the largest, well-powered European datasets available: UK Biobank for hypothyroidism and a large meta-analysis for cirrhosis. We then replicated the key causal effect of hypothyroidism on cirrhosis using an independent set of summary statistics from FinnGen. The replicated direction and significance indicate that our primary conclusion is robust across cohorts with different ascertainment and is not an artifact of a single dataset.

We agree that our study focuses on the contribution of common variants, reflecting both the maturity of current multi-omics methods that operate on GWAS summary data and the availability of large-scale resources. As a result, our conclusions are framed in terms of common-variant architecture. Rare variants and structural variants are incompletely captured by array-based GWAS and likely account for part of the remaining or “missing” heritability. Nonetheless, landmark work shows that the cumulative effect of common variants explains a substantial share of heritability for many human traits (5), and multi-omics methods applied to GWAS summary data are widely validated and have identified credible disease genes in other contexts, such as Alzheimer’s disease (6). Future studies that integrate whole-genome sequencing and structural-variant–aware analyses will be important to quantify additional contributions from rare and complex variation on top of the common-variant effects characterized here.

8. Figure 3B+D: I suggest having both “normal” columns on the same side of each plot to help clarity.

**Response:** Thank you for this helpful suggestion. We have updated Fig. 3B so that the “normal” columns are on the same side across panels, with consistent ordering and legend, improving readability.

**Modification to the Figure:**

9. Figure 4A: I think justification could be given for not having looked at genes that were supported by fewer than 5 criteria. For example, there are 22 genes that were supported by DEG_HT, DEG_LC, WGCNA_HT and WGCNA_LC (not TWAS). Since the TWAS did not, individually, provide evidence for many genes relative to the other methods, there could be some important genes that were missed by the approach taken.

**Response:** Thank you for raising questions regarding the gene-selection approach. In the main text, we adopted a more robust set of selection evidence (i.e., DEG_HT, DEG_LC, WGCNA_HT, WGCNA_LC, and TWAS). The reason is that DEG and WGCNA, which are based on bulk transcriptomic data, are susceptible to confounding factors such as cell-composition differences, inflammatory status, and batch effects, resulting in higher noise and limited specificity (7); in contrast, TWAS integrates eQTL with GWAS to provide a genetic anchoring of gene–disease associations (regulatory signals co-distributed with risk alleles), which can, to some extent, offset bulk-level confounding and improve the robustness and reproducibility of candidate genes (8). Therefore, we required TWAS support as a necessary condition for high-priority genes and set ≥5 lines of evidence as the main-selection threshold.

While this stringent approach ensures the high confidence of our final candidate genes, we also recognize its potential inadequacy in possibly excluding other biologically meaningful genes. In this regard, we are very grateful for your insightful comment. The concern that this strategy may miss important genes is entirely valid and has prompted us to provide a more comprehensive dataset, so that these potential candidates can be represented for the broader research and become a focus of our future investigations. Therefore, we have now incorporated a new supplementary table ***(S7 Table)*** in the Results section. This table provides a complete overview of all combinations derived from the Upset plot, including detailed gene lists for each intersection. By doing so, we not only make the genes you mentioned accessible but also offer a transparent and comprehensive resource for other researchers interested in various intersections among these datasets.

**Modification to the Manuscript:** The details can be seen in lines 274-275, page 16 and S7 Table.

10. PheW-MR analysis: Certain disease traits may be better studied using quantitative measures for a more powerful analysis (e.g. lung function measures such as FEV1 and FVC instead of dichotomising into cases/controls for chronic obstructive pulmonary disease). Were you able to consider quantitative measures in this analysis? If so, why did you exclude them?

**Response:** Thank you for the insightful comment regarding the use of quantitative measures in the PheW-MR analysis. The foundational dataset for our PheW-MR was the comprehensive resource from Zhou et al., which provides harmonized GWAS summary statistics for common disease traits. Our analysis was therefore framed by the case-control data available in this large-scale resource, which did not include quantitative measures for traits like pulmonary function (e.g., FEV1, FVC). We concur that targeted analyses of specific disease families can be enhanced by using quantitative traits, and integrating such data would be a valuable direction for future, more focused investigations building on our work.

11. Supplementary Table 11: I think it would be beneficial to the include associations between HLA-DQ1 and all 675 disease traits, not just those that were statistically significant. Maybe the significant results can be highlighted in some way to make it obvious that they were, indeed, statistically significant. I would also advocate for including the CD27 results for completeness.

**Response:** Thank you for your suggestion regarding Supplementary Table for PheW-MR sections. We have updated the table to include the associations between HLA-DQA1 and all 675 disease traits, rather than limiting it to only statistically significant results. The significant associations are now clearly highlighted to make them easily distinguishable. Additionally, we have included the CD27 results for completeness. Moreover, we have used a random-effects model to estimate the overall effects of the two key genes, HLA-DQA1 and CD27, and these results are now reflected in the revised table.

**Modification to the Manuscript:** The details can be seen in lines 406-411, page 23; lines 869-874, page 44; S14 Table and Supplementary Methods.

**Response to Reviewer 2:**

This manuscript presents a comprehensive assessment of the relationship between hypothyroidism and liver cirrhosis. The study integrates both traditional observational (retrospective cohort) and multi-omics approaches. The use of diverse analytic methods is ambitious and methodologically rich. The identification of HLA-DQA1 and CD27 as immune-related mediators is biologically plausible and translationally relevant. However, several areas require clarification to improve reproducibility and interpretability. Here are some comments to help improve the manuscript:

**Response:** Thank you for your positive feedback and thoughtful suggestions. We appreciate your recognition of the comprehensiveness of our study and the methodological rigor. We also agree that further clarification in certain areas will improve the reproducibility and interpretability of our findings. We will address the following comments to enhance the clarity of the manuscript.

1. The Introduction provides a strong rationale but lacks explicitly stated research questions or objectives. Authors need to add concise sentences at the end of the Introduction section outlining the study-specific objectives.

**Response:** Thank you for your comment. We agree that explicitly stating the research questions and objectives at the end of the Introduction will help to guide the reader and clarify the focus of our study. We have added concise sentences at the conclusion of the Introduction to clearly outline the specific objectives of our study, which include investigating the genetic overlap between hypothyroidism and liver cirrhosis, identifying key immune-related genes, and evaluating potential therapeutic targets.

**Modification to the Manuscript:** The details can be seen in lines 127-131, page 7.

2. The Methods section describes cohort selection, inclusion/exclusion criteria, covariates, and logistic regression modelling. In the Results, authors need to briefly mention (in-text, as Table 1 already notes the variables) the covariates adjusted for in the multivariate analysis to aid reader clarity.

**Response:** Thank you for the suggestion. We agree that reiterating the covariates adjusted for in the multivariable analyses in the Results section will improve clarity. We have now added a detailed description of the covariates adjusted in the analyses and included a complete inclusion/exclusion flow diagram to ensure readers can readily identify this information.

**Modification to the Manuscript:** The details can be seen in lines 174-175, page 9 and line 185, page 12.

3. Figure 1 looks good; however, it will be helpful to provide a figure that captures all the study approaches and shows how the analysis progresses from one level to another (i.e., analytic workflow).

**Response：**Thank you for your suggestion. We have created a new figure that illustrates the complete process for the article, highlighting the various steps and methods used in our analysis.

**Modification to the Figure:**

4. Line 183: authors mention “To further explore potential genetic underpinnings of…” This is the first method applied for genetic assessment in the study, so it is unclear what “further” refers to. Also, please clarify the scale used for the SNP-based heritability estimates; was it on the observed or liability scale?

**Response:** Thank you for your comment. We have revised the text to state that this was the first genetic analysis step. We also clarify that SNP-based heritability is reported on the liability scale. Specifically, we first obtained LDSC estimates on the observed scale and then converted them to the liability scale using disease prevalence from the GBD and published literature. Under this conversion, the Results now report h²_liab as follows: hypothyroidism 17.64% (SE 1.48%) and cirrhosis 12.93% (SE 2.10%).

**Modification to the Manuscript:** The details can be seen in lines 194-197, page 13; lines 199-204, page 13 and lines 705-710, page 36.

5. For the MR analysis, a few issues remain to be clarified:

- The STROBE-MR checklist information and page numbers provided do not correspond to the content in the manuscript or are missing.

- In line with reporting guidelines, it is important to include a schematic diagram summarising the MR design and clearly showing the core assumptions of the method.

- The authors mention using the GWAS Catalog and PhenoScanner V2 to assess exclusion restriction. Please describe how this was done. Importantly, note that PhenoScanner has been offline for an extended period (and the command line version does not appear to work), so clarification is needed here.

**Response:**

1. Thank you for pointing out the issue with the STROBE-MR checklist information. We have reviewed the checklist and corrected any discrepancies between the page numbers provided and the content in the manuscript. We have also ensured that all required information is included in the checklist, and any missing details have been added to align with the manuscript.

2. Thank you for your suggestion regarding the inclusion of a schematic diagram. We agree that a visual representation of the Mendelian randomization (MR) design, clearly showing the core assumptions of the method, will enhance the clarity of the manuscript and align with reporting guidelines. We have added a supplementary figure as a schematic diagram, which summarizes the MR design and highlights the core assumptions, such as the relevance, independence, and exclusion restriction criteria for the instrumental variables.

3. Thank you for pointing out the issue with the incorrect reference to PhenoScanner V2. We sincerely apologize for the oversight. In our data analysis, we referred to Ye X *et al.* (9), which highlighted PhenoScanner V2's powerful ability to remove confounding factors. Based on this, we explored and tried to understand its underlying functionality. However, during the analysis, we found that the tool was no longer operational. To maintain the rigor of our study, we adjusted for potential confounders in the MR analysis using data from the GWAS Catalog instead. Although we were unable to directly use PhenoScanner V2, our methodology was based on its intended use, which led to the misleading reference in the manuscript. We have now corrected this issue and clarified our approach in the revised manuscript.

**Modification to the Manuscript:** The details can be seen in lines 729-738, page 37-38; S1 File and S1 Fig.

6. Authors performed TWAS using OTTERS. It would be helpful to briefly justify the use of OTTERS over other TWAS tools and to discuss the rationale for tissue choice. In particular, please reflect on the implications of using blood-based eQTLs for diseases such as cirrhosis and hypothyroidism.

**Response:** Thank you for the suggestion. We selected OTTERS for two main reasons: data scale and model robustness. Many TWAS methods rely on individual-level reference panels, which limits sample size and statistical power. OTTERS is built for summary-level inputs, allowing us to leverage eQTLGen (N > 31,000) and thereby increase sensitivity to detect candidate risk genes. In addition, the predictive performance of any single model depends on the unknown expression architecture of each gene. OTTERS uses an omnibus strategy that combines four predictors (P+T, lassosum, SDPR, PRS-CS), improving power and stability across both sparse and polygenic architectures. In head-to-head evaluations, OTTERS outperformed single-model approaches such as FUSION in simulations and real-world applications using summary statistics, so it was a suitable and reliable choice for this study.

Regarding tissue choice, we used blood eQTLs from eQTLGen for both statistical and biological reasons. Statistically, the large sample size delivers greater power and more stable effect estimates. Biologically, our question concerns a thyroid–liver axis. Blood is the systemic medium linking these organs, carrying thyroid hormones that regulate hepatic metabolism and transporting circulating immune cells and inflammatory mediators relevant to both hypothyroidism and liver injury. Analyzing gene regulation in blood therefore provides a system-level view of pathways that may mediate inter-organ crosstalk. Evidence from GTEx also indicates that many eQTL effects are shared across tissues, suggesting that variants detected in blood often have concordant effects in other organs (10). We agree that adding high-powered liver and thyroid eQTL resources would yield complementary, tissue-specific insights; at present, such datasets remain limited, and we plan to incorporate them as they become available.

7. The manuscript acknowledges the use of Hashimoto’s thyroiditis data as a proxy and discusses its limitations. Authors may wish to briefly expand on how this may bias immune-related findings in the differential expression analysis.

**Response:** Thank you for this important comment. We agree that using Hashimoto’s thyroiditis (HT) as a proxy for hypothyroidism may bias immune-related differential expression. HT is an autoimmune disease characterized by dense lymphocytic infiltration and a Th1-dominant cytokine milieu (11), so transcriptomic signatures derived from HT tissue may overemphasize immune processes specific to autoimmune attack rather than those shared across all forms of hypothyroidism (12). However, autoimmune thyroiditis is the predominant cause of hypothyroidism in most populations (13), meaning that HT captures the principal biology underlying the majority of clinical cases. In the revision, we clarify this trade-off in the Limitations: HT-based signatures may amplify immune pathways beyond non-autoimmune etiologies, yet they remain a justified proxy for the most common clinical and biological context. Given current constraints on hypothyroidism datasets, we also note our intention to incorporate well-defined hypothyroidism data as they become available to further evaluate generalizability.

**Modification to the Manuscript:** The details can be seen in lines 611-616, page 32.

8. Replication appears to be limited to the cirrhosis component of the analysis. Could there also have been replication for hypothyroidism, or is there a rationale for the current approach? A brief discussion would be helpful.

**Response:** Thank you for the suggestion. We have added an independent replication analysis for hypothyroidism, using GWAS summary statistics that are non-overlapping with the discovery set and phenotype-consistent in definition, and applied the same analytic pipeline as in the discovery stage. The replication showed concordant effect directions and magnitudes and remained statistically significant.

**Modification to the Manuscript:** The details can be seen in lines 209-224, page 13-14; lines 635-640, page 33 and lines 713-717, page37.

9. Authors need to correct typographic errors (e.g., ‘gens’ should be ‘genes’). Please check for similar issues throughout the manuscript and correct them accordingly.

**Response:** Thank you for pointing out the typographic error. We have carefully reviewed the manuscript and corrected the mistake ("gens" to "genes") along with any other similar typographic errors. We have ensured that the entire manuscript is free from such issues.

**Modification to the Manuscript:** The relevant expressions in the text have been revised.

10. Authors need to read through the manuscript line by line to ensure there are no grammatical errors or incomplete statements. For example, in line 112: ‘Building on this…’. it is not clear what ‘this’ refers to. Please avoid such unclear referencing throughout the manuscript.

**Response:** Thank you for your suggestion. We have thoroughly reviewed the manuscript line by line and clarified the use of certain pronouns to ensure there are no grammatical errors or incomplete statements.

**Modification to the Manuscript:** The details can be seen in lines 110-112, page6 and lines 115-116, page 6.

11. The Discussion section could be streamlined to reduce redundancy. There are several places where results are restated unnecessarily. Consider focusing more on interpretation and implications.

**Response:** Thank you for your suggestion. We agree that streamlining the Discussion section will improve clarity and conciseness. We have carefully reviewed the section and removed any unnecessary restatements of the results. Instead, we have focused more on the interpretation of the findings and their broader implications, providing deeper insights into how our results contribute to the understanding of hypothyroidism and liver cirrhosis.

**Modification to the Manuscript:** The relevant expressions in the text have been revised.

12. It appears that the manuscript may have originally been structured with Methods before Results/Discussion. Please go through the current version and ensure that the narrative progresses coherently from Introduction to Results, then Discussion, and that abbreviations used in the Results are defined at first use. For example, some abbreviations appear in the Results but are only defined later in the Methods section.

**Response:** Thank you for your suggestion. We have reviewed the manuscript and made the necessary revisions to ensure the narrative flows coherently from the Introduction to the Results and then to the Discussion. We also re-examined the abbreviations and ensured that those used in the Results section are defined at their first occurrence.

**Modification to the Manuscript:** The relevant expressions in the text have been revised.

**Reference**

1.	Manolio TA, Collins FS, Cox NJ, Goldstein DB, Hindorff LA, Hunter DJ, et al. Finding the missing heritability of complex diseases. Nature. 2009;461(7265):747–53.

2.	Brix TH, Kyvik KO, Hegedüs L. A population-based study of chronic autoimmune hypothyroidism in Danish twins. J Clin Endocrinol Metab. 2000;85(2):536–9.

3.	Loomba R, Schork N, Chen CH, Bettencourt R, Bhatt A, Ang B, et al. Heritability of Hepatic Fibrosis and Steatosis Based on a Prospective Twin Study. Gastroenterology. 2015;149(7):1784–93.

4.	Loh PR, Bhatia G, Gusev A, Finucane HK, Bulik-Sullivan BK, Pollack SJ, et al. Contrasting genetic architectures of schizophrenia and other complex diseases using fast variance-components analysis. Nat Genet. 2015;47(12):1385–92.

5.	Yang J, Benyamin B, McEvoy BP, Gordon S, Henders AK, Nyholt DR, et al. Common SNPs explain a large proportion of the heritability for human height. Nat Genet. 2010;42(7):565–9.

6.	Hu T, Liu Q, Dai Q, Buchman AS, Bennett DA, Tasaki S, et al. Proteome-wide association studies using summary pQTL data of brain, CSF, and plasma identify 30 risk genes of Alzheimer's disease dementia. Alzheimers Res Ther. 2025;17(1):135.

7.	Lähnemann D, Köster J, Szczurek E, McCarthy DJ, Hicks SC, Robinson MD, et al. Eleven grand challenges in single-cell data science. Genome Biol. 2020;21(1):31.

8.	Gusev A, Ko A, Shi H, Bhatia G, Chung W, Penninx BW, et al. Integrative approaches for large-scale transcriptome-wide association studies. Nat Genet. 2016;48(3):245–52.

9.	Ye X, Liu B, Bai Y, Cao Y, Lin S, Lyu L, et al. Genetic evidence strengthens the bidirectional connection between gut microbiota and periodontitis: insights from a two-sample Mendelian randomization study. J Transl Med. 2023;21(1):674.

10.	The GTEx Consortium atlas of genetic regulatory effects across human tissues. Science. 2020;369(6509):1318–30.

11.	Caturegli P, De Remigis A, Rose NR. Hashimoto thyroiditis: clinical and diagnostic criteria. Autoimmun Rev. 2014;13(4-5):391–7.

12.	Luykx JJ, Bakker SC, Visser WF, Verhoeven-Duif N, Buizer-Voskamp JE, den Heijer JM, et al. Genome-wide association study of NMDA receptor coagonists in human cerebrospinal fluid and plasma. Mol Psychiatry. 2015;20(12):1557–64.

13.	Chaker L, Razvi S, Bensenor IM, Azizi F, Pearce EN, Peeters RP. Hypothyroidism. Nat Rev Dis Primers. 2022;8(1):30.
