# Supplementary material for: The role of hypothyroidism in cirrhosis pathogenesis: A retrospective cohort study and multi-omics integration analysis
Source: PLoS Genet. 2025 Nov 7;21(11):e1011947. doi: 10.1371/journal.pgen.1011947 (PMC12611128; doi:10.1371/journal.pgen.1011947)
Supplement: Attachment — Submitted filename: Response_to_Reviewers_auresp_2.docx [file pgen.1011947.s021.docx]

**Response to Reviewer 1：**

Thank you to Dr Zhou and colleagues for addressing my prior concerns. I have no further suggestions to make.

**Response:** We sincerely thank you for your positive feedback and for acknowledging our revisions. We are also deeply grateful for your time and the insightful suggestions you provided throughout the entire review process. Your valuable comments were instrumental in strengthening our work, leading to a qualitative leap in the manuscript. To formally express our immense gratitude for your dedication and pivotal role in this process, we will be including a special acknowledgement of the reviewers' contributions in the manuscript.

**Response to Reviewer 2:**

Thank you for responding to my comments. I have two minor comments.

**Response:** We thank you again for taking the time from your busy schedule to review our response. We greatly appreciate the two valuable additional comments you have provided, which are crucial for us to further enhance the rigor of our manuscript. The quality of our paper has been substantially elevated thanks to your diligent and insightful feedback. As a token of our sincere appreciation for this invaluable contribution, we will be including a formal acknowledgement of the reviewers' efforts in our manuscript.

We have provided detailed responses to your comments one by one below for your review.

1. In response to one of the reviewer 1's comments, authors stated that their revised analysis is “more robust” because the SNP-based heritability was converted to the liability scale, which they suggested influences the genetic correlation (rg). However, the liability-scale adjustment applies to heritability estimates (h²) rather than to rg. Thus, converting to the liability scale does not improve or correct rg estimation. If the updated rg (0.2135) differs from the preliminary value (0.1566), it likely reflects differences in input data, QC parameters, or model specification rather than the liability transformation. Additionally, if gencov intercept is constrained, it could also affect rg estimates, especially when sample overlap is not fully accounted for. The authors should clarify what they did here.

**Response:** We sincerely thank you for your exceptionally meticulous and insightful review. Your astuteness in questioning the discrepancy between the preliminary and final genetic correlation estimates is entirely correct, and we are grateful for your rigorous attention to detail, which has significantly enhanced the accuracy of our manuscript.

We deeply apologize for the confusion and the lack of clarity in our previous submission. Your query prompted a thorough internal audit. This audit confirmed that the change in rg was not related to the liability-scale conversion, as you reasonably queried. The discrepancy stemmed from an internal version control oversight early in our project. The preliminary value (0.1566) was an exploratory finding from an unfinalized dataset, which was regrettably and mistakenly included in an early draft due to a communication lapse. We had, in fact, identified and rectified this data inconsistency ourselves during our first major revision to ensure all analyses were based on the final, consistent GWAS summary statistics. The final reported rg of 0.2135 is the definitive result.

We are profoundly grateful for your diligence, which has allowed us to clarify this matter and correct the record. To ensure full transparency and our commitment to reproducible science, our detailed analysis scripts and logs can be immediately available upon request by contacting the corresponding author. Thank you once again for your invaluable contribution. Your rigorous scrutiny has not only helped us correct this important detail but has also directly contributed to improving our internal quality control processes.

2. There was heterogeneity in the MR analysis, which would suggest horizontal pleiotropy, even with pleiotropy test suggesting otherwise (a common scenario in MR). To address this situation, authors need to check through their instruments and remove any of them associated with the outcome variable at p < 0.05. Lets see what outcome you have thereafter, but I would expect at least the heterogeneity would be gone. Please, also report the p values, both for outcome and exposure, for all the instruments used (probably as a supplementary table) for transparency.

**Response:** We are very grateful for your highly insightful and constructive feedback. Your astuteness in highlighting the heterogeneity (*P* = 0.04) in our initial analysis as a key area for deeper examination is greatly appreciated. We share your valid concern that heterogeneity may signal underlying horizontal pleiotropy. At the same time, we considered the possibility that this heterogeneity could stem from vertical pleiotropy, a phenomenon not inconsistent with the fundamental assumptions of MR (1, 2). Indeed, our findings from both the original MR-Egger test and the newly incorporated MR-PRESSO analysis lend strong credence to this interpretation, as neither test detected significant directional pleiotropy.

Regarding your suggestion to remove instruments associated with the outcome at *P* < 0.05, we appreciate the intention to apply a stringent filter. However, we were concerned that this threshold might be overly restrictive and could inadvertently remove valid instruments exhibiting vertical pleiotropy, which, as noted by Burgess S *et al.*, "could lead to distorted causal estimates" (3). Therefore, to address your concern, we adopted the same stringent filtering threshold used in our manuscript for the GWAS Catalog screen to filter instruments against the outcome GWAS summary statistics data. Any IVs associated with the outcome exceeding this threshold were removed.

Furthermore, we have integrated a cascade of analytical steps to bolster our findings. These enhancements include applying Steiger filtering to confirm causal directionality (4); quantifying heterogeneity with the I^2^ statistic alongside Cochran's Q test to guide our use of fixed or random-effects IVW models (5, 6); and incorporating the MR-PRESSO method as an additional powerful tool for detecting and correcting for pleiotropic outliers (7). Finally, to bring our evidence to a comprehensive conclusion, we conducted a meta-analysis to synthesize the estimates from our discovery and replication cohorts, providing a powerful summary of the overall causal effect (8, 9).

In complete alignment with your excellent suggestion for enhanced transparency, we have now included a comprehensive **Supplementary Table S4**. This table details all harmonised IVs used in the final analysis, including their alleles, effect estimates, standard errors, and *P*-values for both the exposure and the outcome. We are confident that these extensive revisions, prompted entirely by your thoughtful guidance, have substantially strengthened the manuscript. We are profoundly grateful for your invaluable contribution, which has elevated the quality and reliability of our work.

**Modification to the Manuscript:** The details can be seen in lines 215-228, page 14-15, lines 733-759, page 38-39 and **Supplementary Table S4-5**.

**Reference**

1.	Sanderson E, Glymour MM, Holmes MV, Kang H, Morrison J, Munafò MR, et al. Mendelian randomization. Nat Rev Methods Primers. 2022;2.

2.	Cho Y, Haycock PC, Sanderson E, Gaunt TR, Zheng J, Morris AP, et al. Exploiting horizontal pleiotropy to search for causal pathways within a Mendelian randomization framework. Nat Commun. 2020;11(1):1010.

3.	Burgess S, Davey Smith G, Davies NM, Dudbridge F, Gill D, Glymour MM, et al. Guidelines for performing Mendelian randomization investigations: update for summer 2023. Wellcome Open Res. 2019;4:186.

4.	Hemani G, Tilling K, Davey Smith G. Orienting the causal relationship between imprecisely measured traits using GWAS summary data. PLoS Genet. 2017;13(11):e1007081.

5.	Higgins JP, Thompson SG, Deeks JJ, Altman DG. Measuring inconsistency in meta-analyses. Bmj. 2003;327(7414):557–60.

6.	Bowden J, Del Greco MF, Minelli C, Davey Smith G, Sheehan N, Thompson J. A framework for the investigation of pleiotropy in two-sample summary data Mendelian randomization. Stat Med. 2017;36(11):1783–802.

7.	Verbanck M, Chen CY, Neale B, Do R. Detection of widespread horizontal pleiotropy in causal relationships inferred from Mendelian randomization between complex traits and diseases. Nat Genet. 2018;50(5):693–8.

8.	Gao H, Zheng S, Yuan X, Xie J, Xu L. Causal association between inflammatory bowel disease and 32 site-specific extracolonic cancers: a Mendelian randomization study. BMC Med. 2023;21(1):389.

9.	Jackson D, White IR, Riley RD. Quantifying the impact of between-study heterogeneity in multivariate meta-analyses. Stat Med. 2012;31(29):3805–20.
